# Recurrent Hematocolpos and Hematometra as a Late Complication of Endometrial Ablation: A Case Report

**DOI:** 10.7759/cureus.97624

**Published:** 2025-11-24

**Authors:** Fatima Hameed, Rabeet Hassan, Asiya Alvi

**Affiliations:** 1 Obstetrics and Gynecology, Bolton NHS Foundation Trust, Manchester, GBR; 2 Pediatric Endocrinology, Indus Hospital Network, Lahore, PAK

**Keywords:** cyclical abdominal pain, heavy menstrual bleeding, hematocolpos, radiofrequency ablation, usg-guided hematometra drainage

## Abstract

Endometrial ablation functions as a minimally invasive procedure to treat heavy menstrual bleeding when other treatments prove ineffective. The procedure maintains safety for most patients, yet they may experience delayed complications, which include obstructive issues such as hematometra and hematocolpos. We present the case of a 48-year-old patient who developed recurring symptomatic hematocolpos and hematometra three months following her endometrial ablation procedure for treating excessive menstrual bleeding. The patient experienced periodic lower abdominal discomfort, which imaging tests, including pelvic transvaginal ultrasound, CT scan, and MRI, confirmed resulted in hematometra. The patient underwent two drainage procedures under general anesthesia. The patient underwent a hysterectomy after a third occurrence of identical symptoms through a shared decision-making process. The histopathological examination revealed intrauterine fibrosis and cervical canal blockage, but no evidence of cancer. This case illustrates an unusual yet crucial complication that occurs following endometrial ablation treatments. Medical staff must recognize cyclic pelvic pain and amenorrhea as warning indicators of obstructive pathology to provide timely treatment. The management of this condition requires a systematic treatment approach that starts with observation and pain management before advancing to hysteroscopic revision. The first step in managing obstructive pathology requires complete imaging through transvaginal ultrasound and MRI when needed to assess the degree of anatomical blockages. The initial approach for treating obstructive pathology involves monitoring the patient while providing pain management through conservative methods. The treatment plan shifts to hysteroscopic revision as a minimally invasive procedure after conservative approaches fail to deliver sustained results. The patient needs a hysterectomy as their last treatment option after both conservative and hysteroscopic methods fail to deliver symptom relief. The selection of surgical treatment depends on how severe the symptoms are and how long they persist, as well as the extent of fibrosis and anatomical blockage severity, and the patient’s desire to keep their uterus. Medical practitioners need to evaluate post-ablation hematometra or hematocolpos in women who present with cyclic pelvic pain and amenorrhea. The prevention of morbidity requires immediate imaging tests along with prompt medical interventions.

## Introduction

The medical procedure of endometrial ablation aims to help women who have completed their childbearing years and suffer from abnormal uterine bleeding. The procedure uses ablation to eliminate functional endometrial tissue, which results in reduced or complete cessation of bleeding. The available endometrial ablation techniques include rollerball resection, thermal balloon, microwave, and radiofrequency ablation methods. Endometrial ablation procedures demonstrate high initial success rates, yet patients can develop late-onset endometrial ablation failure (LOEAF), which emerges several months to multiple years after treatment completion [1,2].

The symptoms of LOEAF include persistent or recurring bleeding and pelvic pain during cycles and obstructed menstruation because of intrauterine fibrosis or cervical stenosis. The blockage of menstrual flow results in blood accumulation within the uterus (hematometra) and vagina (hematocolpos), which leads to discomfort [3,4]. The condition requires immediate detection for successful diagnosis and treatment outcomes.

In this case, we present a patient who required multiple drainage procedures before undergoing a hysterectomy because endometrial ablation resulted in recurring hematocolpos and hematometra. The report details the diagnostic process and treatment options for this rare complication that occurs after ablation procedures. The incidence of hematometra after endometrial ablation is generally low, estimated at 1% to 3% of patients, making it a rare yet very important complication.

## Case presentation

A 48-year-old woman visited the gynecological unit with lower abdominal pain and amenorrhea three months after undergoing endometrial ablation to treat her persistent heavy menstrual bleeding. The patient suffered from five years of unresponsive menorrhagia despite hormonal therapy and antifibrinolytic treatment. The patient underwent NovaSure radiofrequency ablation without complications during her postoperative recovery period.

The patient experienced cyclic suprapubic pain during her menstrual periods three months after the procedure. She experienced abdominal cramps that spread to her back while experiencing pelvic fullness without any vaginal discharge or bleeding. She had no history of cervical surgery, pelvic infections, or uterine procedures. The patient had no other health issues and did not use blood thinners as medication. The patient’s vital signs remained normal during the assessment. The abdominal examination showed minimal suprapubic discomfort but no signs of peritoneal irritation. The cervix showed a normal health status. The bimanual examination showed a firm, mobile, enlarged uterus with mild tenderness. The blood test results and serum beta-human chorionic gonadotropin measurements showed normal values.

**Figure 1:**
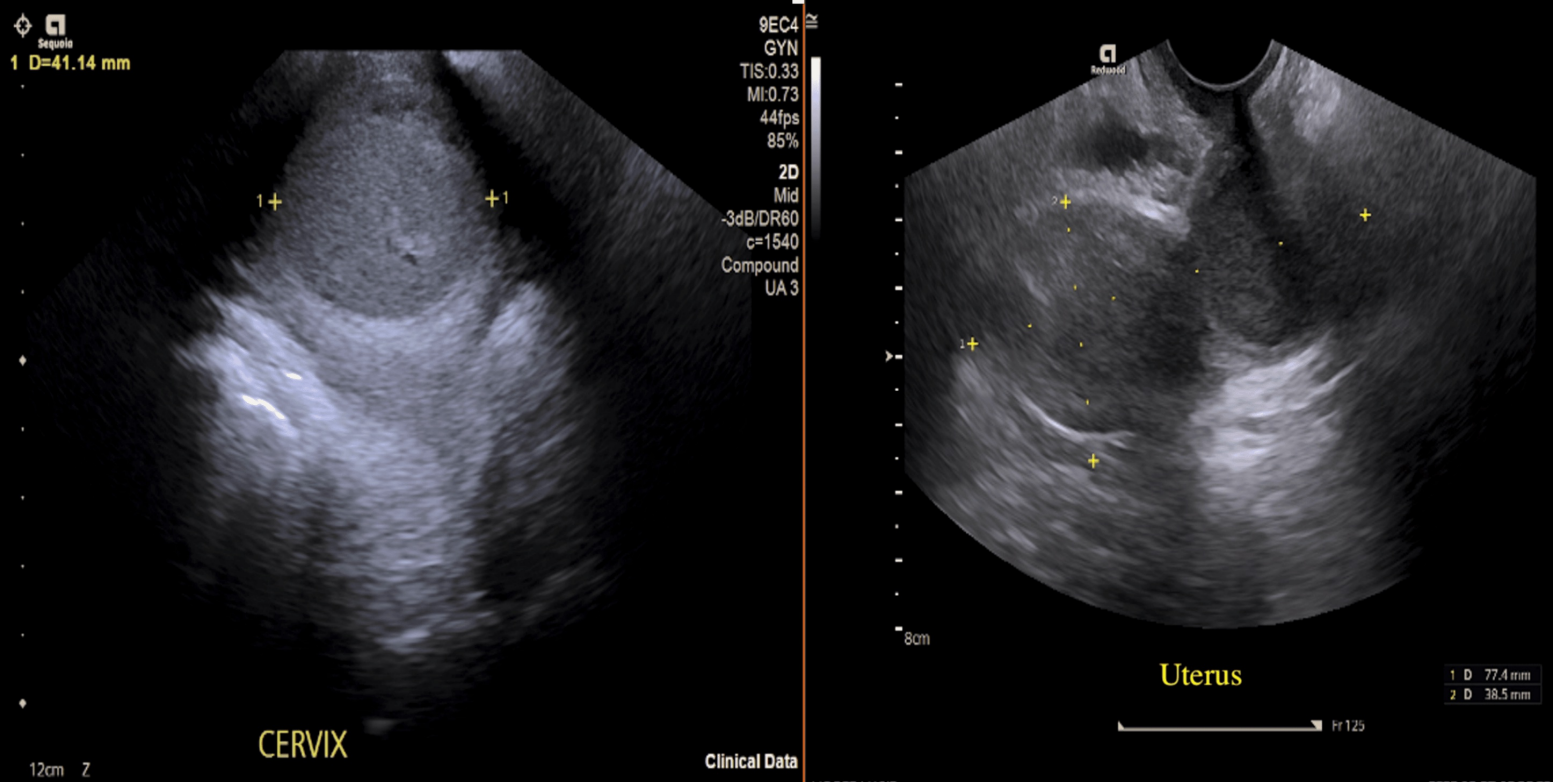
Transvaginal ultrasound of the pelvis showing the uterus and cervix. The anteverted uterus measures 79 × 34 mm. Endometrial thickness is 16 mm, and it is distended by homogeneous matter. Within the lower uterus/cervix, there is a homogeneous collection measuring 34 × 38 × 41 mm. No increase in vascularity can be noted. Appearances in keeping with hematometra/hydrometrocolpos.

The transvaginal ultrasound showed a 7.9 × 3.4 cm fluid-filled uterine cavity with heterogeneity, which matched hematometra, and a 3.5 × 2.8 cm fluid collection in the vaginal vault that indicated hematocolpos. The ultrasound examination showed that the cervix had an elongated shape, while the canal section remained invisible (Figure 1). The ovaries, together with the adnexa, showed normal findings. The procedure under general anesthesia involved cervical dilation followed by suction evacuation of 120 mL of dark blood from the uterine cavity. The uterine cavity received ultrasound guidance during the curettage procedure to remove all remaining tissue. The patient was discharged on the following day after showing no symptoms.

**Figure 2:**
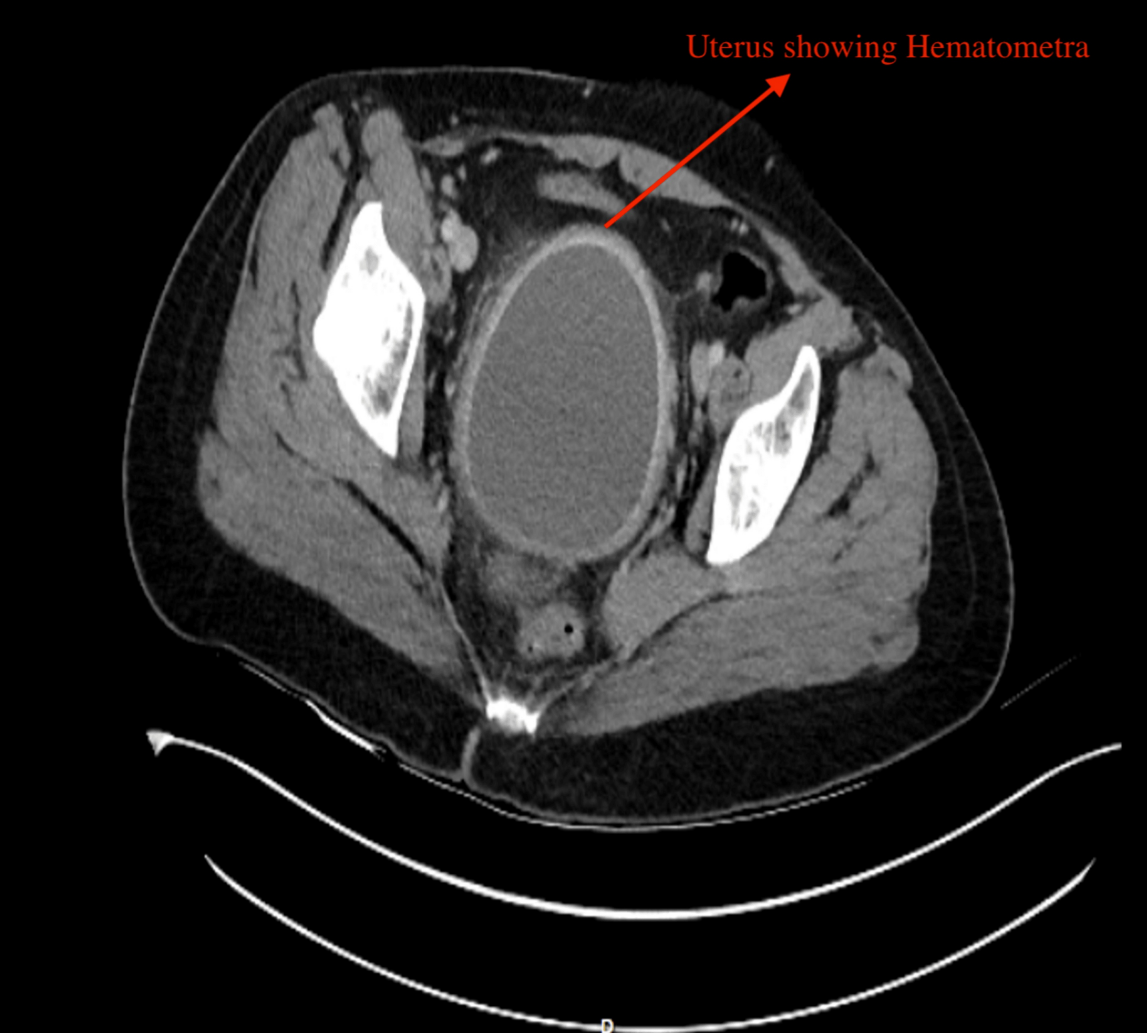
CT of the abdomen and pelvis with contrast showing hematometra. Marked increase in the uterine volume (measuring 15.5 × 9 × 9 cm in the craniocaudal, anteroposterior, and transverse diameters) and thickness of the endometrial wall containing fluid measuring 20 HU in density. There is no evidence of gas locule to suggest infection.

The patient returned two months later because of recurring pain symptoms. The ultrasound results showed echogenic fluid inside the uterus, which indicated hematometra. The pelvic MRI showed that the uterine cavity was expanded with blood and fibrotic tissue, while the cervical canal remained blocked from the vagina. The CT showed a marked increase in the uterine volume (measuring 15.5 × 9 × 9 cm in the craniocaudal, anteroposterior, and transverse diameters) and thickness of the endometrial wall containing fluid measuring 20 HU in density. There was no evidence of a gas locule to suggest infection (Figure 2). The adnexa and vaginal vault were normal. Another drainage procedure was performed for the patient. The patient received information about treatment options for recurrence, but decided to maintain a conservative approach at that time.

**Figure 3:**
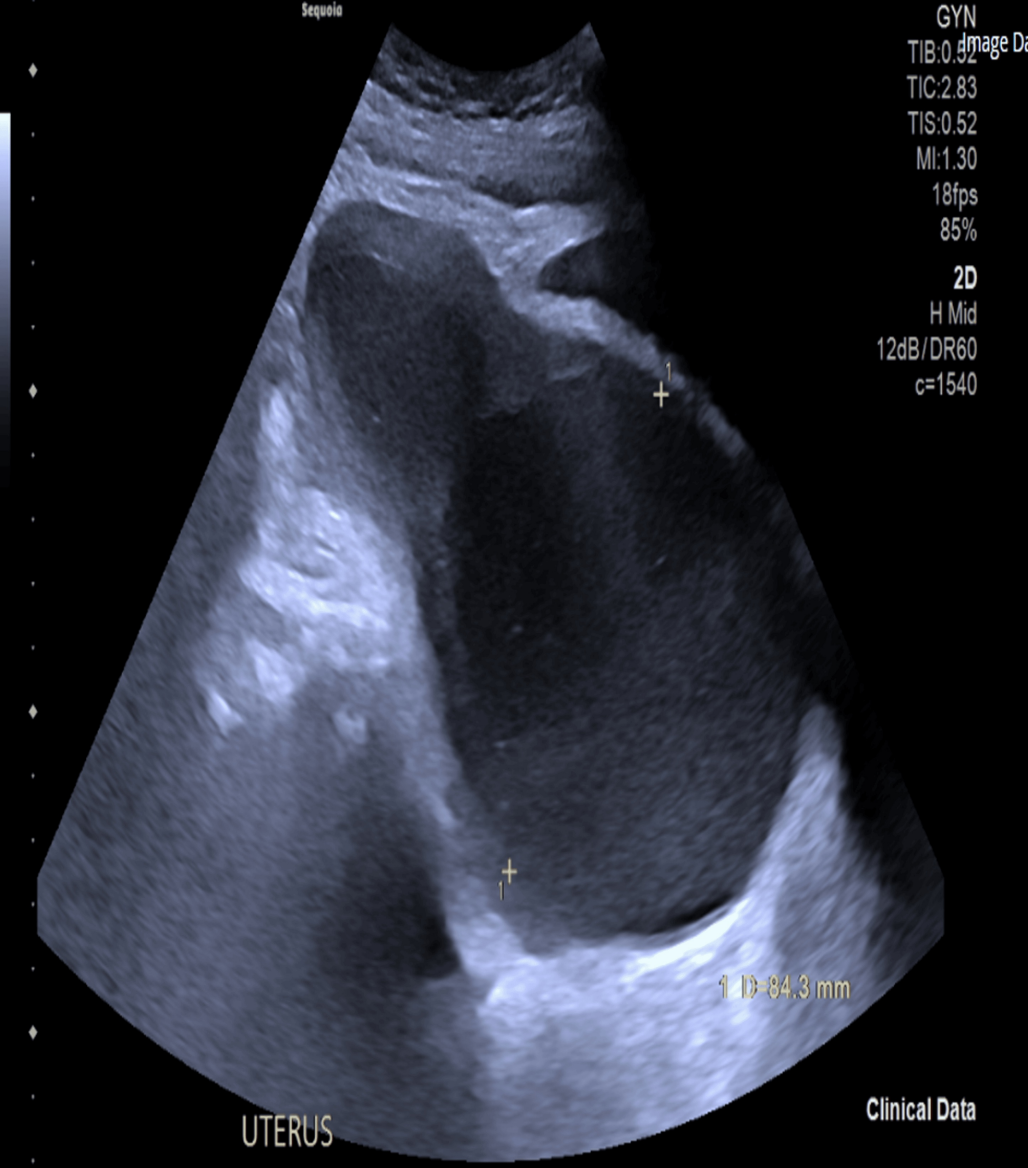
Repeat pelvic ultrasound on third presentation showing hematometra. Again, the cavity is distended by 17.5 mm and contains homogeneous matter. The cervix contains a homogeneous collection measuring 31 × 29 × 25 mm, with no vascularity seen. The uterus measured 8.4 × 7.2 cm  in size.

The patient experienced pelvic pressure together with pain during the second two-month period. The imaging results showed hematometra together with probable cervical fibrosis. A repeat pelvic ultrasound showed an anteroverted uterus measuring 8.4 × 7.2 cm in size, containing a homogenous collection measuring 31 × 29 × 25 mm (Figure 3). The patient underwent a total abdominal hysterectomy because of multiple recurring episodes. The uterus displayed increased tension while containing 150 mL of blood clots. The examination revealed dense fibrosis together with complete blockage of the cervical canal. The histological examination revealed endometrial scarring with trapped endometrial tissue, but no cancerous cells were present.

The patient experienced no complications during her postoperative period, which led to her discharge on day four after surgery. The patient showed no symptoms during her six-month follow-up appointment.

## Discussion

Endometrial ablation leads to two uncommon complications, which block blood flow from the uterus, resulting in hematometra and hematocolpos. The blood accumulation occurs because endometrial tissue becomes trapped behind the obstruction. The blood flow obstruction occurs because fibrosis produces stenotic areas in the cervical region and lower uterine segment that still experience menstrual cycle changes [5,6]. The blood accumulation leads to LOEAF, which causes recurring bleeding, obstructed menses, and pelvic pain [7,8].

The condition produces mild pelvic discomfort during menstrual periods without any signs of bleeding. Patients who stop menstruating after ablation surgery often believe their pelvic discomfort stems from different medical conditions. Healthcare providers should evaluate hematometra as a possible cause when ablation surgery patients present with cyclic pelvic pain or fullness [9]. The uterine cavity contains echogenic material that causes distension according to transvaginal ultrasound, but MRI and CT provide superior visualization of adhesions, cornual sequestration, and hematosalpinx [10].

This patient required two drainage procedures because endometrial ablation creates uterine scarring that leads to restenosis according to established medical knowledge [3,5]. The medical literature shows that cervical dilatation gives short-term pain relief, but studies confirm it fails to create lasting patency because most patients develop multiple blockages, according to multiple case reports and review articles [1]. The first step in treating this condition involves performing imaging tests, which include transvaginal ultrasound and pelvic MRI to identify physical blockages and adhesions according to expert guidelines [5,11]. The combination of hysteroscopic resection of intrauterine adhesions under ultrasound guidance in multiple retrospective and prospective studies demonstrates successful uterine patency restoration with preserved reproductive anatomy [1,11].

The treatment plan should proceed based on operative laparoscopy results, which assess both safety and success rates by examining synechiae and fibrosis distribution [11]. The established relapse criteria enable healthcare providers to monitor patient re-obstruction indicators following treatment [12]. The medical literature supports hysterectomy as the definitive treatment for patients who have failed hysteroscopic and conservative therapies because it provides effective symptom management with minimal recurrence [12]. Healthcare providers must reveal all potential delayed complications from endometrial ablation procedures to patients during their first consultation. The risk of obstruction decreases when doctors perform preoperative assessments and protect the internal os during surgery and conduct postoperative checks. The recommended post-ablation follow-up schedule includes an initial check-up during the first three months, followed by six-month assessments for two years to monitor new pelvic pain, amenorrhea, and pelvic fullness symptoms. The follow-up process should include transvaginal ultrasound tests to detect early signs of hematometra and hematocolpos. Patients require an MRI scan when their ultrasound results remain ambiguous or their symptoms continue. Healthcare providers must create individualized treatment plans for patients that present all available options while respecting their personal treatment preferences.

## Conclusions

Endometrial ablation results in rare yet dangerous complications, which include recurrent hematometra and hematocolpos expansion and cyclic pain. Clinical assessment should be performed for post-ablation patients who develop new cyclic pelvic pain. The conditions develop because scarring and stenosis block menstrual flow, which results in uterine or amenorrhea. The definitive treatment for recurrent cases includes hysterectomy, but cervical dilatation and drainage provide temporary pain relief. The evaluation of alternative treatments should begin before patients choose a hysterectomy if they wish to preserve their uterus. The treatment plan should include hysteroscopic adhesiolysis and other minimally invasive procedures to enable doctors and patients to make decisions together. The prevention of long-term complications needs immediate surgical intervention, together with proper patient education and early detection of complications.
